# Simultaneous detection of influenza A, B and respiratory syncytial virus in wastewater samples by one-step multiplex RT-ddPCR assay

**DOI:** 10.1186/s40246-024-00614-8

**Published:** 2024-05-20

**Authors:** Anastasia Zafeiriadou, Lazaros Kaltsis, Nikolaos S. Thomaidis, Athina Markou

**Affiliations:** Laboratory of Analytical Chemistry, Department of Chemistry, National and Kapodistrian University of Athens, 15771 ZografouAthens, Greece

**Keywords:** Influenza A, Influenza B, Respiratory Syncytial Virus, Wastewater, RT-ddPCR

## Abstract

**Background:**

After the occurrence of the COVID-19 pandemic, detection of other disseminated respiratory viruses using highly sensitive molecular methods was declared essential for monitoring the spread of health-threatening viruses in communities. The development of multiplex molecular assays are essential for the simultaneous detection of such viruses even at low concentrations. In the present study, a highly sensitive and specific multiplex one-step droplet digital PCR (RT-ddPCR) assay was developed for the simultaneous detection and absolute quantification of influenza A (IAV), influenza B (IBV), respiratory syncytial virus (RSV), and beta-2-microglobulin transcript as an endogenous internal control (IC B2M).

**Results:**

The assay was first evaluated for analytical sensitivity and specificity, linearity, reproducibility, and recovery rates with excellent performance characteristics and then applied to 37 wastewater samples previously evaluated with commercially available and in-house quantitative real-time reverse transcription PCR (RT-qPCR) assays. IAV was detected in 16/37 (43%), IBV in 19/37 (51%), and RSV in 10/37 (27%) of the wastewater samples. Direct comparison of the developed assay with real-time RT-qPCR assays showed statistically significant high agreement in the detection of IAV (kappa Cohen’s correlation coefficient: 0.834, p = 0.001) and RSV (kappa: 0.773, p = 0.001) viruses between the two assays, while the results for the detection of IBV (kappa: 0.355, p = 0.27) showed good agreement without statistical significance.

**Conclusions:**

Overall, the developed one-step multiplex ddPCR assay is cost-effective, highly sensitive and specific, and can simultaneously detect three common respiratory viruses in the complex matrix of wastewater samples even at low concentrations. Due to its high sensitivity and resistance to PCR inhibitors, the developed assay could be further used as an early warning system for wastewater monitoring.

**Supplementary Information:**

The online version contains supplementary material available at 10.1186/s40246-024-00614-8.

## Background

The co-circulation of different types of respiratory viruses in communities and the importance of their surveillance was demonstrated even before the emergence of SARS-CoV-2. Surveillance of influenza A (IAV), influenza B (IBV) and respiratory syncytial viruses (RSV) is a priority for global health systems to assess the burden of infections and prevent future outbreaks at an early stage due to the significant risks they pose to public health [1]. Respiratory diseases have severely jeopardized people’s health and caused heavy economic losses, while influenza virus has been present in almost every seasonal outbreak each year, leading to 290.000–650.000 deaths [2]. The Covid-19 pandemic has affected the infection dynamics of non-SARS-CoV-2 respiratory viruses and a further need has arisen to identify the periods of their altered seasonality [1].

Nowadays, various diagnostic tests are used to detect respiratory pathogens, including molecular tests and antigen detection tests [3]. Antigen tests can rapidly detect respiratory viral antigens [4–7], but with varying diagnostic accuracy between tests and moderate sensitivity compared to molecular tests [8]. Quantitative reverse transcription polymerase chain reaction (RT-qPCR) tests are widely used for the detection and quantification of pathogenic viruses in clinical [9, 10] or highly complex samples, such as wastewater samples [11–14]. However, several factors can lead to inaccurate and unreliable results due to the limitations of RT-qPCR technology [15, 16], resulting in the implementation of ddPCR as an alternative approach. The main principle of ddPCR is to divide the PCR reaction mixture into several smaller, independent partitions following the detection of fluorescence at the end of the PCR amplification. The distribution of targets in the partitions follows a Poisson distribution and the quantification is performed using established statistical models and without the need for calibration curves. Overall, ddPCR is a highly sensitive method for the detection of target molecules at very low concentrations and is insensitive to inhibitors due to its partitioning into different droplets and partial reactions [17].

The application of molecular assays in highly complex samples is challenging, due to the inhibition in PCR reactions. Wastewater represents a complex matrix rich in inhibitor compounds that often interfere with the PCR amplification. However, the monitoring of viruses in wastewater has proven to be a valuable tool to determine the spread of disease and infection dynamics in real time [18]. Several strategies have been used to overcome inhibition in environmental samples [15, 19]. One widely used approach is the use of inhibitor removal reagents, which often increase the cost of analysis, or dilution of samples, an approach that may result in cost-effective dilution of PCR inhibitors, but also reduces the sensitivity of the assay and leads to false negative results [20, 21]. Therefore, evaluating the performance of ddPCR assays that are resistant to inhibitors and may not require inhibitor-tolerant reagents or sample dilution should be a priority in the concept of wastewater-based epidemiology.

The prevalence of viral coinfections has not yet received sufficient attention. Co-infection of patients with various respiratory viruses must be recognized immediately so that this high-risk group can be treated in time. It is also necessary to monitor the circulation patterns of viruses in the population to support public health policy measures. The development and analytical validation of highly sensitive and specific multiplex RT-ddPCR assays is essential for simultaneous baseline monitoring of respiratory viruses even at low concentrations, especially when complex matrices such as wastewater need to be analyzed.

In the present study, a highly sensitive and specific one-step multiplex RT-ddPCR assay was developed and analytically validated for the simultaneous detection and absolute quantification of Influenza A, B and RSV, together with the detection of a specific region of the beta-2-microglobulin (B2M) transcript as an endogenous internal control (IC). The developed assay was also applied in wastewater samples from the Attica region to evaluate its potential for detection and quantification of the targeted viruses and to directly compare the performance of the newly developed assay with RT-qPCR assays available in the laboratory. The inclusion of an endogenous internal control helps to evaluate the performance of the sampling and experimental workflow, while limiting false negative results.

## Materials and methods

### Wastewater sampling

24-h composite flow proportional raw wastewater samples were collected at the Wastewater Treatment Plant (WWTP) of Attica, the region of Greece that includes the greater Athens area and its suburbs. 37 raw wastewater samples were collected and total nucleic acids were extracted as previously described [22, 23]. Wastewater samples were collected in pre-cleaned 1L high-density polyethylene bottles and transported to the laboratory at 4 °C, where they were processed immediately upon arrival. Biosafety guidelines were followed during sample collection, transport, and analysis.

### Multiplex One-step RT-ddPCR

A single-step multiplex RT-ddPCR assay was developed and analytically validated for simultaneous detection and absolute quantification of influenza A M gene (matrix protein), influenza B NS gene (nonstructural protein), RSV M gene (matrix protein), and beta-2 microglobulin (B2M) as an internal control. B2M gene mRNA was used as an endogenous control as it is expressed in all nucleated cells and is a human biomarker of good stability and with minimal degradation over 24-h in wastewater samples [24, 25]. The inclusion of an endogenous internal control is very important in the developed assay as it is essential for evaluating the accurate sampling and helping in limiting the number of false negative results [26]. The selections of primer and probe sequences are based on CDC for Influenza A and B, whereas for RSV and B2M are based on our previous studies [13, 26] (Additional file 1: PCR primers and probes sequences, Table S1). All hydrolysis probes were designed with a 6-carboxyfluorescein (FAM) or hexachlorofluorescein (HEX) fluorophore and ZEN/Black Hole Quencher 1 (BHQ1) quenchers for more efficient quenching (Integrated DNA Technologies, USA).

Multiplex one-step RT-ddPCR was performed in the QX600™ Droplet Digital™ PCR System (Bio-Rad, USA). All preparation steps for the ddPCR setup were performed in a dedicated pre-PCR room and a PCR Hood dedicated for the separation of ddPCR reactions to avoid contaminations. The developed ddPCR assay was performed using One-step RT-ddPCR Advanced kit for Probes (Bio-Rad, USA) and the reaction mix consisted of 5.0 μl of Supermix, 2.0 μl of Reverse Transcriptase, 1.0 μl of 300 mM dithiothreitol (DTT), 2 μl of primer pairs and TaqMan hydrolysis probes mix, 5 μl of RNA template and H_2_O to a final volume of 20 μl. Primer/probe sets for IAV and IBV were prepared at a final concentration of 900 nM (1x) and 300 nM (1x), respectively, while for RSV and IC B2M the final concentrations were 450 nM (0.5x) and 150 nM (0.5x), respectively. By adding one FAM and HEX assay at 1 × concentration and another FAM and HEX assay at 5 × concentration, an upper and a lower cluster were formed in the same well, resulting in four clearly separated clusters in a 2D scatter plot and allowing multiplexing of the developed assay.

PCR was performed in the C1000 Touch™ Thermal Cycler (50 °C/1 h for the reverse transcription step, 95 °C/10 min, 40 cycles of 94 °C/30 s and 60 °C/1 min and a final step at 98 oC/10 min). A temperature ramp of 2 °C/s was set on all PCR steps. 96-well plate was read in the QX600 Droplet Reader (Bio-Rad, USA) and the absolute copy number of the four targets was calculated using the QuantaSoft analysis software (Bio-Rad, USA), according to the Poisson Distribution. Positive and negative controls were used in each run to evaluate the performance of the assay.

### Synthetic DNA standards

Three synthetic DNA oligonucleotides were developed and used for analytical validation of the assay (gBlocks; Integrated DNA Technologies, USA). Each of these synthetic oligonucleotides contained the target sequences of IAV, IBV, and RSV, including primer and probe binding sites.

Inactivated influenza A/B and respiratory syncytial virus (Helix Elite™ Molecular Standard, Microbiologics, USA) was used to evaluate the analytical specificity and recovery of the developed assay. The standard contains an inactivated viral pellet with copies of all three viruses detected in the multiplex assay. After resuspension of the pellet in the appropriate amount of H2O, RNA extraction was performed using the “QIAamp® Viral RNA Mini Kit” (Qiagen, Germany).

## Results

### Optimization of annealing temperature

**Fig. 1:**
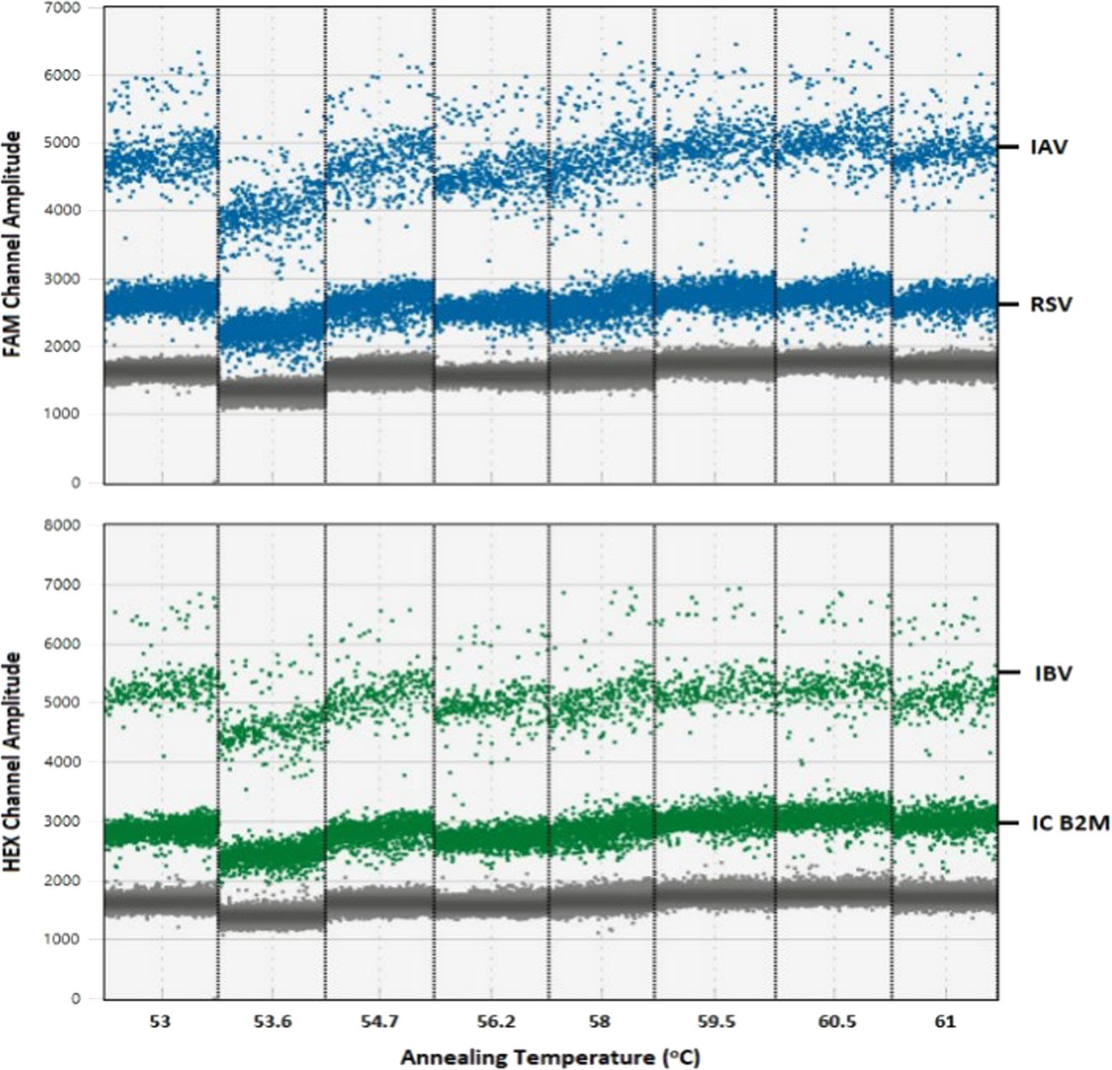
Amplification 1-D plots from the thermal gradient optimization experiment. Annealing temperatures from 53 to 61 °C were tested. FAM^high^: Influenza A, FAM^low^: RSV, HEX^high^: Influenza B, HEX^low^: Internal Control Beta-2-Microglobulin (IC B2M)

The annealing temperature is one of the decisive factors influencing the specificity of ddPCR. To optimize the annealing temperature, multiplex RT-ddPCR was performed over a temperature gradient of 53–61 °C. According to our results, 60 °C was the best option as it yielded well-separated droplet clusters with minimal volumes of "rain" for each virus type and was used for subsequent ddPCR experiments (Fig. 1).

### Analytical validation

Synthetic oligonucleotides for each virus type (gBlocks; Integrated DNA Technologies, USA) and the commercially available inactivated viral pellet containing copies of influenza A, B, and RSV viruses (Helix Elite™ Molecular Standards, Microbiologics, USA) were used for analytical validation of the developed assay (10^3^ copy number/μl). RNA derived from the MCF-7 cell line, where only B2M is expressed was also used for the control’s preparation. Synthetic oligonucleotides for the three viruses were mixed at equal copy number concentrations and used to estimate the limit of blank (LOB), limit of detection (LOD), limit of quantitation (LOQ), analytical sensitivity, and linear dynamic range (LDR), while the Helix Elite™ Molecular Standard was used to estimate analytical specificity, intra- and inter-assay repeatability, and analytical recovery.

### Analytical specificity

**Fig. 2:**
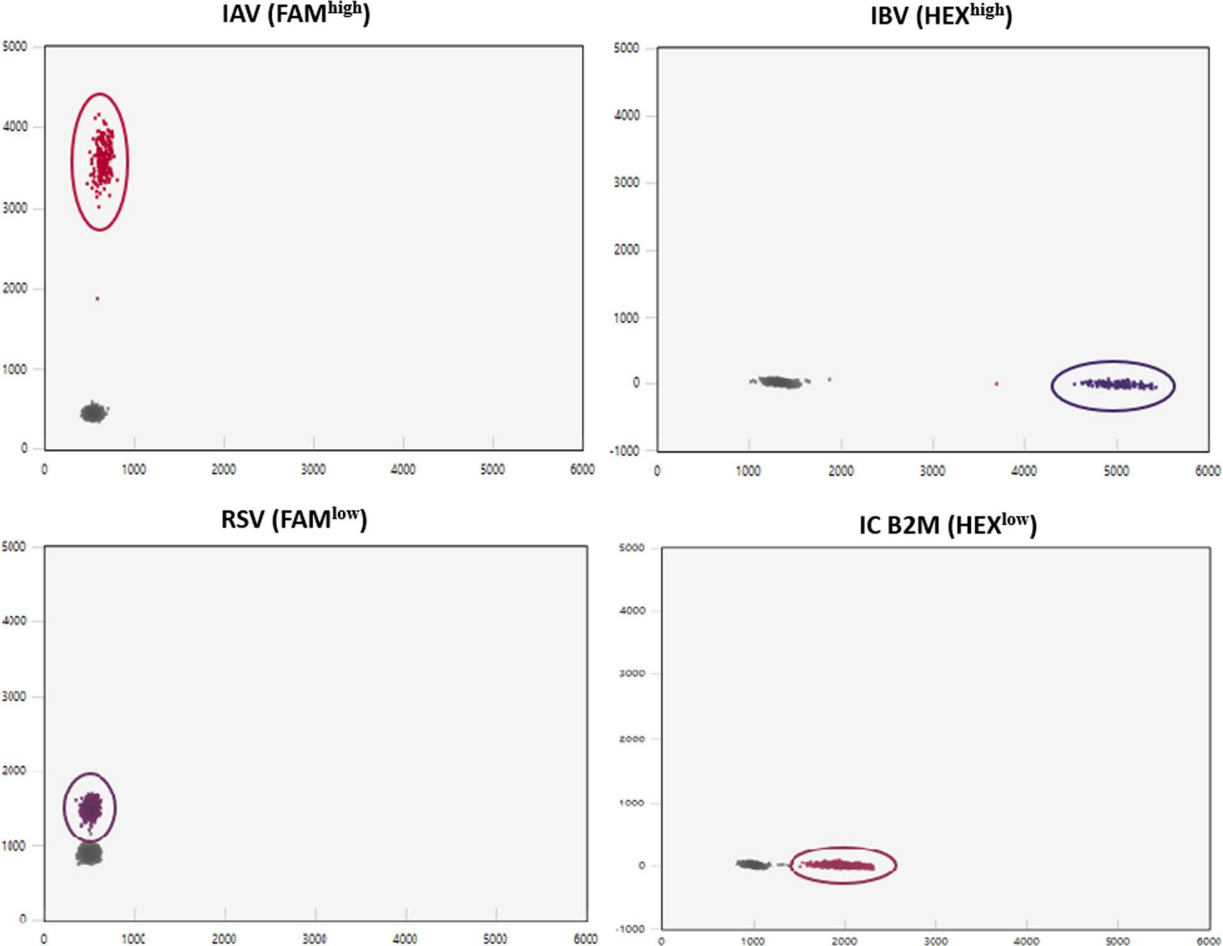
Analytical specificity of the developed one-step multiplex RT-ddPCR assay. Four different 2-D plots representing each target in the four individual assays

To evaluate the analytical specificity of the developed assay, RNA from the Helix standard and RNA from the MCF-7 cell line containing copies of internal control B2M were mixed at equal concentrations to produce a control sample for the evaluation of analytical specificity (Control A). Each individual ddPCR reaction contained primers and probes for a specific virus type in the absence of all others, resulting in the detection of a specific target transcript in the four individual reactions of the multiplex RT-ddPCR. Subsequently, multiplex RT-ddPCR was performed in one step in the presence of all primers and probes for all virus types. According to our results, the analytical specificity was excellent, as only one droplet cluster was obtained that corresponded to the specific primer/probe pair added in each individual run (Fig. 2).

### Linear dynamic range

**Fig. 3:**
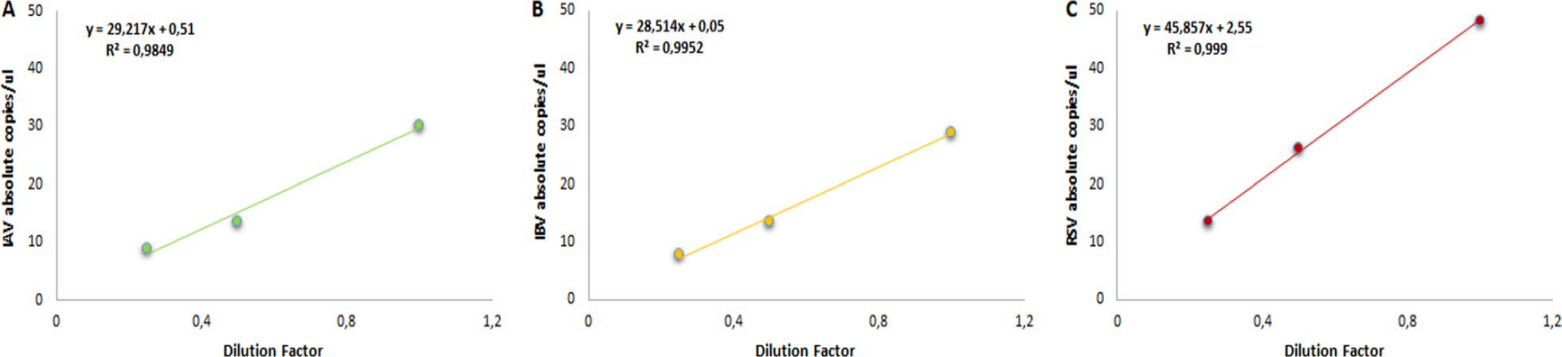
Linear dynamic range of the developed assay for **A** IAV, **B** IBV and **C** RSV

For linear dynamic range assessment, known concentrations of synthetic oligonucleotides of each virus type were mixed at the same copy number concentration. The LDR of each primer/probe set targeting all three viruses was assessed by serial 1:2 dilutions of approximately 100 copies/μl to 25 copies/μl of the sample input, with all reactions performed in triplicate. The results are presented in a linear regression plot showing the absolute number of copies/μl (Y-axis) versus the dilution factor (X-axis) (Fig. 3). The correlation coefficients (R2) were 0.9849, 0.9952, and 0.999 for influenza A, influenza B, and RSV, respectively, indicating a precise linear relationship (Fig. 3).

### Analytical sensitivity

LOB, LOD, and LOQ of the multiplex RT-ddPCR assay were defined as described elsewhere [27, 28]. For LOB determination, RNA was used from 12 wastewater samples selected from a period when respiratory viruses were not detected in the community according to their seasonal pattern. For each virus type, LOB was defined as the highest copy number concentration found in the wastewater samples according to the following Eq. (1):

1 $$LOB = mean \left( {copy\;number\;blank} \right){ } + { }1.645xSD\left( {copy\;number\;blank} \right){ }$$

The number of “false-positive” ddPCR droplets defining LOB was 0–2 for IAV and RSV and 0–1 for IBV. The LOB was set at 0.09, 0.05, and 0.17 absolute copies/μl of ddPCR for influenza A, B, and RSV, respectively, while any ddPCR result with a value below the LOB was reported as “not detected”." All effluent samples were positive for IC B2M, indicating that the concentration and extraction steps were successful.

For LOD evaluation, synthetic oligonucleotides of each virus and RNA obtained from an MCF-7 cell line with known copies were mixed at equal concentrations (control B) after serial tenfold dilutions at four different concentrations (100, 20, 10, and 1 absolute copies/μl) to determine the LOD and LOQ of the developed multiplex assay. 100 copies/μl were run in triplicate, while the lower concentrations were run 7 times. LOD was defined as the lowest copy number concentration that could be distinguished from LOB with > 95% confidence (Table 1), according to the following Eq. (2):

2 $$LOD = LOB + 1,645xSD\left( {low\;concentration\;copy\;number\;sample} \right)$$

**Table 1:** Evaluation of LOD and LOQ of the developed one-step multiplex RT-ddPCR assay

Virus type	Control B (copies/μl of input)	Mean values of absolute copies/μl (ddPCR)	SD	CV%
**IAV**	100	25.2	1.72	6.8
	20	3.8	0.47	12.3
	10	2.2	0.4	18.4
	1	0.2	0.16	77.5
**IBV**	100	24.5	1.91	7.8
	20	4.0	0.66	16.4
	10	2.3	0.62	27.6
	1	0.2	0.11	69.5
**RSV**	100	69.5	4.08	5.9
	20	10.7	1.32	12.3
	10	5.6	1.07	18.9
	1	0.6	0.36	56.6

The lowest detected concentration with a CV ≤ 25(LOQ) is represented in bold

Each virus was detected at all concentrations, but with variable repeatability depending on the concentration level. According to Eq. (2), the LOD value was set at 0.4 copies/μl ddPCR reaction for influenza A (or 1.4 copies/μl RNA sample input), 0.2 copies/μl ddPCR reaction for influenza B (or 0.9 copies/μl RNA sample input), and 0.8 copies/μl ddPCR reaction (or 3 copies/μl RNA sample input) for RSV.

LOQ was set as the lowest detected concentration that had a CV ≤ 25 and was set at 2.2 copies/μl for IAV, 4 copies/μl for IBV and 5.6 copies/μl of ddPCR reaction for RSV, or 8.7, 16.1 and 22.6 copies/μl of RNA sample input, respectively (Table 1).

### Analytical sensitivity: comparison between singleplex ddPCR and a multiplex ddPCR assay

Multiplex assays must be well optimized to maintain or improve the analytical sensitivity of their singleplex counterparts. In this experiment, the LOD of singleplex real-time PCR assays targeting each virus is compared to an equivalent panel of an evolved multiplex ddPCR assay. The efficiency and viral copies of the multiplex RT-ddPCR assay were compared with singleplex assays targeting each virus individually. The results showed that the two methods were comparable (Table 2).

**Table 2:** Comparison of copies/μl between the newly developed multiplex assay and singleplex assays

Virus type	Copies/μl	
	Singleplex	Multiplex
IAV	62.8	58
IBV	41.6	42.8
RSV	286	228
IC B2M	572	544

### Intra- and inter-assay repeatability

Intra-assay repeatability was evaluated using Control B at the same concentrations that were used to evaluate LOD and LOQ. CV% ranged from 6.8 to 77.5% for IAV, 7.8 to 69.5% for IBV and 5.9 to 56.6% for RSV, with CV% presenting high values at the very low concentrations of each virus (Table 1).

Inter-assay repeatability was determined by evaluating the undiluted Control A in six different runs on six different days (Table 3).

**Table 3:** Inter-assay repeatability of Control A

Virus type	Average copies/μl	SD	CV%
IAV	19.1	3.0	15.7
IBV	12.1	2.3	19.1
RSV	62.7	6.3	10.1
IC B2M	37.5	5.7	15.1

### Analytical recovery

Analytical recovery of the developed assay for each virus was determined by spiking known concentrations of the Helix Elite™ Molecular Standard (10^3^ and 10^4^copies/reaction) in two individual aliquots of the same wastewater sample (Table 4). Samples were run in triplicate. Analytical recoveries (%) for each virus were estimated based on the following Eq. (3) described elsewhere [22]:

3 $$Recovery\left( {\text{\% }} \right) = \frac{copies\;of\;spiked\;sample - endo\;genous\;copies\;of\;waste\;water\;sample}{{theoretical\;copies\;that\;were\;spiked\left( {pure\;sample} \right)}}{*}100{ }$$

**Table 4:** Analytical Recovery of the developed assay using the Helix Molecular Standard at two different concentrations

Virus type	Spiked concentration	Found copies/μl Mean value (SD)	Recovery% Mean value (range, CV%)
RSV	10^4^copies/reaction	115 (9.5)	86.8 (79.5–93.9, 8.3%)
	10^3^copies/reaction	11.5 (0.29)	92.6 (91.3–95.3, 2.5%)
IAV	10^4^copies/reaction	29.4 (5.84)	77.2 (59.6–88.3, 19.9%)
	10^3^copies/reaction	3.1 (0.36)	91.1 (94.3–100, 11.9%)
IBV	10^4^copies/reaction	19.7 (0.61)	85.2 (82.3–87.5, 3.1%)
	10^3^copies/reaction	1.8 (0.45)	83.6 (62.6–100, 26%)

The pure sample corresponds to the same copy number concentration spiked in nuclease-free water, eliminating the influence of inhibitors that might be present in the wastewater sample.

### Direct comparison study of the developed multiplex one-step RT-ddPCR assay with commercially available IVD and in-house RT-qPCR assays

**Fig. 4:**
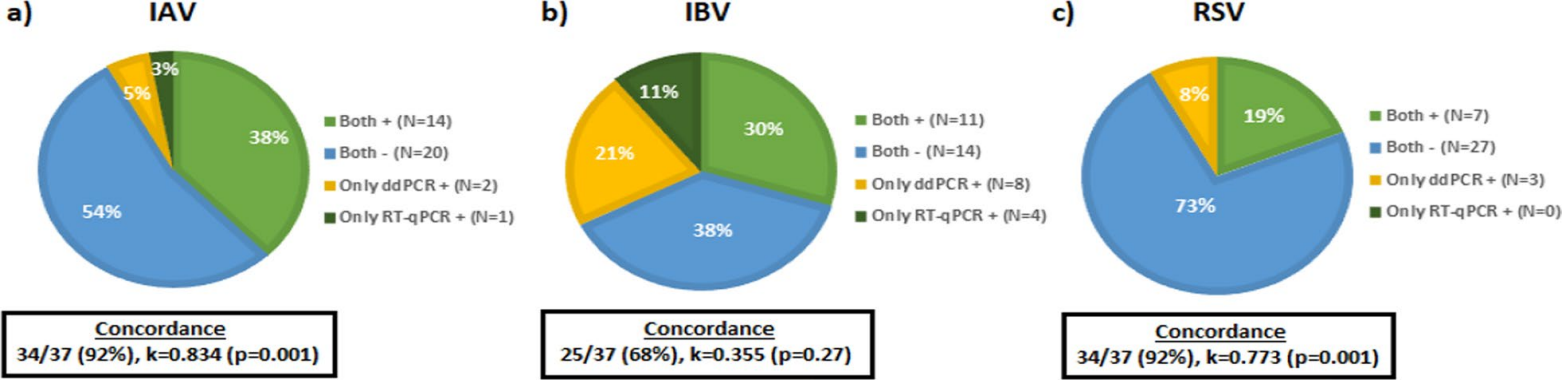
Comparison between the newly developed RT-ddPCR assay and RT-qPCR assays for 37 wastewater samples

The newly developed one-step multiplex RT-ddPCR assay was directly compared with a commercially available IVD RT-PCR assay (Bio-Rad Reliance SC2/FluA/FluB RT-PCR kit, Bio-Rad, USA) for the detection of IAV and IBV viruses and with an RT-qPCR assay for the detection of RSV previously developed in our laboratory [13]. The same 37 wastewater samples were analyzed by both methods, and their correlation was assessed using Cohen’s kappa test (SPSS Statistics 26.0, IBM Corp, Armonk, USA). According to our results, the agreement between the two tests was 92% for IAV and RSV, while IBV appeared to have a low agreement (62%). It is important to note that according to our results, a number of samples were only detected using the newly developed RT-ddPCR assay (Fig. 4).

### Quantification of influenza A, B and RSV in wastewater samples

After analytical validation, the developed one-step multiplex RT-ddPCR assay was used for the detection and quantification of IAV, IBV and RSV in 37 raw wastewater samples from the Attica region. According to our newly developed RT-ddPCR results, 16/37 (43%), 19/37 (51%) and 10/37 (27%) samples were found positive for IAV, IBV and RSV, respectively. The average copy number/L was 2.1 × 10^4^, 6.9 × 10^3^, and 1.9 × 10^4^ for IAV, IBV, and RSV, respectively. All samples were positive for internal control B2M (mean: 1.3 × 10^6^ copies/L), demonstrating that the concentration and extraction steps were successful.

## Discussion

The detection of respiratory viruses is of great importance given the significant risk they pose to human health. The potential of wastewater genomic surveillance for early detection and the importance of monitoring viruses for disease management at the community level is emphasized. Infection dynamics can be monitored using RT-qPCR assays and NGS, but these methods are subject to some limitations. The reliance on establishing a standard curve, the differences in protocols, reagents, instrumentation and data analysis and interpretation between laboratories, and the influence of inhibitors present in highly complex samples that can affect the efficiency of amplification have led to the development and adoption of droplet digital PCR (ddPCR) methods [16, 29, 30]. In this study, a highly sensitive and specific one-step multiplex RT-ddPCR assay was developed and validated for the simultaneous detection of influenza A, influenza B and RSV. The newly developed RT-ddPCR assay has been evaluated and can be successfully used in wastewater samples, a complex matrix containing a large number of inhibitors that often interfere with the amplification and efficiency of real-time RT-qPCR tests and lead to false negative results.

To date, several mainly single-plex RT-ddPCR assays have been developed for the detection and quantification of influenza viruses and RSV in both clinical [31–33] and wastewater samples [34–38]. Boehm et al. [39, 40] developed multiplex RT-ddPCR assays for a number of respiratory viruses, including influenza A, B and RSV, and efficiently quantified their viral loads in the population through wastewater surveillance. Our results also demonstrate the importance and potential of developing a sensitive, specific and less inhibition-prone RT-ddPCR assay capable of simultaneously detecting viruses through wastewater surveillance to provide important complementary data on the circulation of health-threatening viruses in communities. It is important to note that many of the RT-ddPCR assays described to date have not included an endogenous internal RNA control. This is a crucial factor for sample evaluation and assay quality, contributing to more reliable virus detection and limiting false-negative results.

In our newly developed assay, no influence of inhibitors was detected in undiluted samples. Detection of virus in the same undiluted samples was not possible when analyzed with the RT-qPCR assay, and a tenfold dilution was required to detect viral load, increasing the cost of the analysis. A number of samples analyzed with both assays (RT-qPCR and RT-ddPCR) proved positive only with the newly developed RT-ddPCR assay. This finding could be due to the high sensitivity of the ddPCR assay and its resistance to inhibitors, which is consistent with other studies that have found better performance of ddPCR compared to RT-qPCR [29]. However, in the case of IAV and IBV, only one and four samples, respectively, were detected with the Bio-Rad Reliance SC2/FluA/FluB IVD kit, likely due to the higher sample volume required per PCR reaction.

Here we show that our newly developed RT-ddPCR is highly sensitive as it can detect the viruses with only half the sample volume in the PCR reaction and is more reliable due to the inclusion of an endogenous internal control that tests the pre-analytical steps. It is also consistent with the RT-qPCR methods routinely used in daily practice. We believe that the assay exemplifies the power of multiplex RT-ddPCR assays and will be a valuable tool for the detection and absolute quantification of respiratory viruses, even when a large number of inhibitors are present in the sample.

## Conclusions

Α highly sensitive and specific one-step multiplex RT-ddPCR test for the simultaneous detection of influenza A, influenza B and RSV has been developed and validated. The newly developed RT-ddPCR assay has been evaluated and can be successfully used in wastewater samples, a complex matrix containing a large number of inhibitors that often interfere with the amplification and efficiency of real time RT-qPCR assays, leading to false negative results. We believe that the present study will provide important information on the advantages and superiority of ddPCR compared to RT-qPCR assays in the testing of wastewater samples, an area where there are a few studies on multiplex RT-ddPCR assays. Here we show that our newly developed assay can be used for monitoring common circulating viruses in wastewater and can detect these specific viruses simultaneously and even at low concentrations, without the need to dilute samples and with little influence of inhibitors.

### Supplementary Information

**Additional file 1**. PCR primers and probes sequences.
