# Supplementary material for: Simultaneous detection of influenza A, B and respiratory syncytial virus in wastewater samples by one-step multiplex RT-ddPCR assay
Source: Hum Genomics. 2024 May 20;18:48. doi: 10.1186/s40246-024-00614-8 (PMC11103825; doi:10.1186/s40246-024-00614-8)
Supplement: Supplementary file 1 — Additional file 1. PCR primers and probes sequences. [file 40246_2024_614_MOESM1_ESM.docx]

**Supplementary material**

**Supplementary Table**

**Table S1.** PCR primers and probes sequences.

| Gene |  | Sequence 5’🡪3’ |
| --- | --- | --- |
| M gene Influenza A | **Forward primer** | CAAGACCAATCYTGTCACCTCTGAC |
|  | **Reverse primer** | GCATTYTGGACAAAVCGTCTACG |
|  | **Probe** | TGCAGTCCTCGCTCACTGGGCACG |
| NS gene Influenza B | **Forward primer** | TCCTCAAYTCACTCTTCGAGCG |
|  | **Reverse primer** | CGGTGCTCTTGACCAAATTGG |
|  | **Probe** | CCAATTCGAGCAGCTGAAACTGCGGTG |
| M gene RSV | **Forward primer** | GGCAAATATGGAAACATACGTGA |
|  | **Reverse primer** | CTTTTTCTAGGACATTGTATTGAACAG |
|  | **Probe** | CTGTGTATGTGGAGCCTTCGTGAAG |
| B2M transcript | **Forward primer** | GCCTGCCGTGTGAACCATGT |
|  | **Reverse primer** | AAATGCGGCATCTTCAAACCTC |
|  | **Probe** | CATGATGCTGCTTACATGTCTCGATCCCAC |
